# Supplementary material for: Identification of the alternative sigma factor regulons of Chlamydia trachomatis using multiplexed CRISPR interference
Source: mSphere. 2023 Sep 25;8(5):e00391-23. doi: 10.1128/msphere.00391-23 (PMC10597470; doi:10.1128/msphere.00391-23)
Supplement: Figure S1 — RT-qPCR analysis of lepA. [file msphere.00391-23-s0002.pdf]

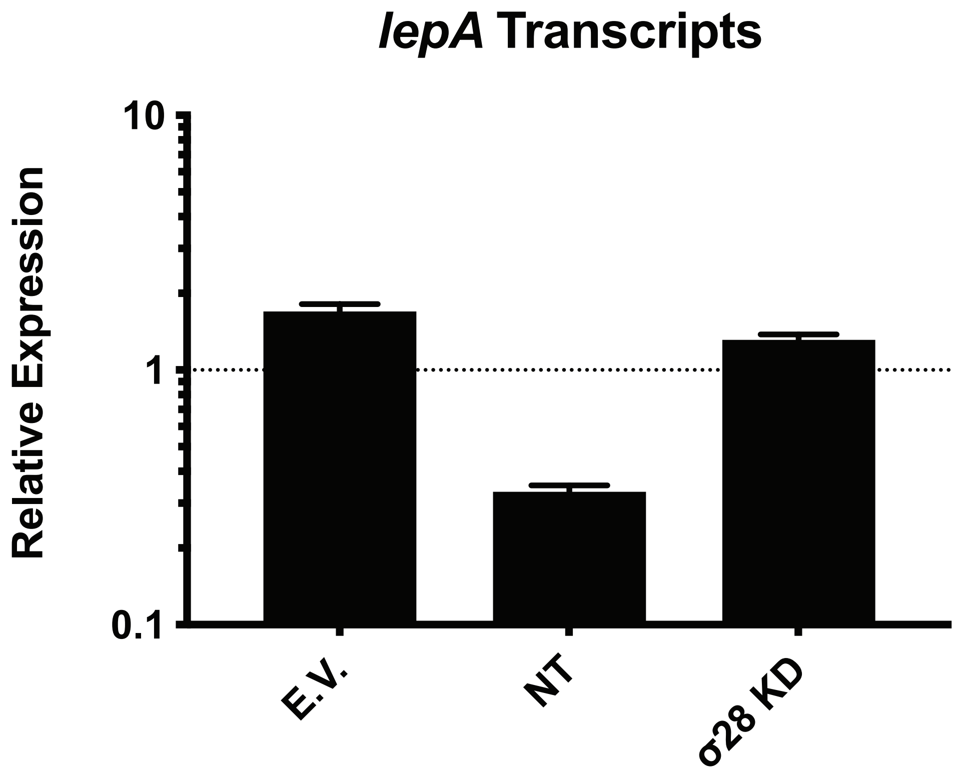

Supplemental Figure S1. RT-qPCR analysis of *lepA*. Knockdown strains (x-axis) were induced or not with 10 nM aTc at 4 hpi. Samples were collected at 24 hpi and processed as described in the methods. Uninduced ratios of cDNA:16S are set to 1, induced samples' cDNA:16S ratios are shown as a relative proportion. E.V.: Empty vector, dCas12 only with no crRNA. NT: Non-Targeting. All samples include two biological replicates.
