# Supplementary material for: Identification of the alternative sigma factor regulons of Chlamydia trachomatis using multiplexed CRISPR interference
Source: mSphere. 2023 Sep 25;8(5):e00391-23. doi: 10.1128/msphere.00391-23 (PMC10597470; doi:10.1128/msphere.00391-23)
Supplement: Table S1 — Plasmids, strains, and primers. [file msphere.00391-23-s0003.pdf]

Supplemental Table S1. List of Plasmids, Strains, and Primers used in the study.

Construct Plasmid	Relevant genotype	Ori	Source of Reference
pBOMBL::L2	*bla* Ptet::*mCherry*	pUC19	(1)
pBOMBL-σ^{28}_10xH::L2	*bla* Ptet::*fliA_10xH* (σ^{28})	pUC19	This study
pBOMBL-σ^{54}_10xH::L2	*bla* Ptet::*rpoN_10xH* (σ^{54})	pUC19	This study
pBOMBL-σ^{66}_10xH::L2	*bla* Ptet::*rpoD_10xH* (σ^{66})	pUC19	This study
pBOMBL-σ^{28}_FLAG-σ^{54}_10xH::L2	*bla* Ptet::*fliA_FLAG* (σ^{28})- *rpoN_10xH* (σ^{54})	pUC19	This study
pBOMBL12-*As_ddCpf1vaa*::L2 (pL12CRia(e.v.)::L2)	*bla* P_{tet}::*As_ddCpf1vaa*	pUC19	(1)
pL12CRia(*non-targeting*)::L2	*bla* P_{dnaKm}:: *non-targeting*_crRNA P_{tet}:: *As_ddCpf1vaa*	pUC19	(2)
pL12CRia(σ^{28})::L2	*bla* P_{dnaKm}:: σ^{28}*-targeting*_crRNA P_{tet}:: *As_ddCpf1vaa*	pUC19	This study
pL12CRia(σ^{54})::L2	*bla* P_{dnaKm}:: σ^{54}*-targeting*_crRNA P_{tet}:: *As_ddCpf1vaa*	pUC19	This study
pL12CRia(σ^{28}/σ^{54})::L2	*bla* P_{dnaKm}:: σ^{28}/σ^{54}*-targeting*_crRNAs P_{tet}:: *As_ddCpf1vaa*	pUC19	This study
pL12CRia(σ^{28}/σ^{54}/*incA*)::L2	*bla* P_{dnaKm}:: σ^{28}/σ^{54}/*incA-targeting*_crRNAs P_{tet}:: *As_ddCpf1vaa*	pUC19	This study

E. coli Strain	Relevant genotype	Source of Reference
DH10β	*Δ(ara-leu) 7697 araD139 fhuA ΔlacX74 galK16 galE15 ϕ80dlacZΔM15 (e14-) recA1 relA1 endA1 nupG rpsL* (Str^{R}) *rph spoT1 Δ(mrr-hsdRMS-mcrBC)*	New England BioLabs

Primer name	Sequence	Features	Usage
16S rRNA	TAAAGAAGCACCGGCTAACTC	Forward qPCR primer	For qPCR of *16S rRNA*
16S rRNA	AACTTACCTTTCCGCCTACAC	Reverse qPCR primer	For qPCR of *16S rRNA*
fliA F	GGGTACCACGCAGTGTTTAT	Forward qPCR primer	For qPCR of *fliA*
fliA R	CACAAAGTTCTCCATCAGTAGGT	Reverse qPCR primer	For qPCR of *fliA*
rpoN F	ACAGAAACTCTATCCGCATACC	Forward qPCR primer	For qPCR of *rpoN*
rpoN R	GGGAGAGGTTGCCTACAATATAC	Reverse qPCR primer	For qPCR of *rpoN*

rpoD F	GATCGCGCAGTATCTCATCA A	Forward qPCR primer	For qPCR of *rpoD*
rpoD R	TGGGCAGAAGATTAAGGAA GTG	Reverse qPCR primer	For qPCR of *rpoD*
incA F	TCTGATCGCTCCACAAATCA C	Forward qPCR primer	For qPCR of *incA*
incA R	CTTCTCTTTGCAGATCCTGG TATA	Reverse qPCR primer	For qPCR of *incA*
lepA F	CCTGTAGTGTTTGCTGGTAT CT	Forward qPCR primer	For qPCR *lepA*
lepA R	ATCGTAAGAGCTGAGTCGTT TAG	Reverse qPCR primer	For qPCR *lepA*
sigma28/(pBOMBL)/5'	tcttcacacaggacatctgcatgaagactcac gatctcg		For amplification of *fliA_10xH* into pBOMBL (EagI/KpnI digested)
sigma28_10xH /(pBOMBL)/3'	tttgaatggtcgaccggtacctaatggtgatgg tgatggtgatggtgatggtgaagcagactgga caatgtac		For amplification of *fliA_10xH* into pBOMBL (EagI/KpnI digested)
sigma54/(pBOMBL)/5'	tcttcacacaggacatctgcatgttgcatcagc atcaaac		For amplification of *rpoN_10xH* into pBOMBL (EagI/KpnI digested)
sigma54_10xH /(pBOMBL)/3'	tttgaatggtcgaccggtacctaatggtgatgg tgatggtgatggtgattgtggatagtatgtcga gaattctctg		For amplification of *rpoN_10xH* into pBOMBL (EagI/KpnI digested)
sigma 28_FLAG/(sigma54)/3'	aacaattctcctacttgtcgtcatcgtctttgtag tcaagcagactggacaatgtac	Used with sigma28/(pBOMBL)/5'	For amplification of *fliA_FLAG* as a transcriptional fusion with *rpoN_10xH* into pBOMBL (EagI/KpnI digested)
sigma 54/(sigma28_FLAG)/5'	gactacaaagacgatgacgacaagtaggag aattgttatgttgcatcagcatcaaac	Used with sigma54_10xH/(pBOMBL)/3'	For amplification of *rpoN_10xH* as a transcriptional

			fusion with *fliA_FLAG* into pBOMBL (EagI/KpnI digested)
sigma66/(pBOMBL)/5'	tcttcacacaggacatctgcatgcgcatggatacgctag		For amplification of *rpoD_10xH* into pBOMBL (EagI/KpnI digested)
sigma66_10xH/(pBOMBL)/3'	tttgaatggtcgaccggtacctaatggtgatggtgatggtgatggtgatggtgatttttataactttta atcttacccgaac		For amplification of *rpoD_10xH* into pBOMBL (EagI/KpnI digested)
sigma54_crRNA/(sigma28)/5' overlap	agtagaaattaTAACCTATGTTGCATCAGCATATCTACAAGAGTAGAAATTA	Anneal to sigma54_crRNA/(incA)/3'overlap	To create crRNA for *rpoN* for multiplexed (x3) knockdown; use with *incA* and σ^{28} multiplex crRNA gBlocks
sigma54_crRNA/(incA)/3'overlap	*AAGTTCTCTGAAA*TAATTTCTACTCTTGTAGATATGCTGATGCAACATAGGTTA	Anneal to sigma54_crRNA/(sigma28)/5'overlap	To create crRNA for *rpoN* for multiplexed (x3) knockdown; use with *incA* and σ^{28} multiplex crRNA gBlocks
Bam Flank Left/(pBOMBL12CRia)/5'	tgaatataattttaattatatcacgcactagctca		To amplify multiplexed (x3) crRNA for insertion into BamHI-digested pBOMBL12CRia (e.v.)::L2 plasmid
Bam Flank Right/(pBOMBL12CRia)/3'	tgtgaaagtgggtcttaagacgtcg		To amplify multiplexed (x3) crRNA for insertion into BamHI-digested pBOMBL12CRia (e.v.)::L2 plasmid

gBlock Name	Sequence	Features	Usage
σ^{28} crRNA	tgtgaaagtgggtcttaagacgtcggtactgcatgtgacgcacgtagatcatgca*TTCACCGGTGGAGACGGTTTTCTTATAATGACACC*TAATTTCTACTCTTGTAGAT**CAAACTAAAAAATAAGGCTAA**CAAATAAAACGAAAGGCTCAGTCGAAAGACTGGGCCTTTCGTTTTATcaacagcggtctactgaatctgagctagtgcgtgatataattaaaattatattca	Lower case for plasmid overlap and spacer, *italicized* for P_{dnaK} sequence, underlined for crRNA scaffold, **bold** for σ^{28} targeting sequence, Upper case for *rrnB*1 terminator	For CRISPRi knockdown of σ^{28}; insert into BamHI-digested pBOMBL12CRia (e.v.)::L2 plasmid
σ^{54} crRNA	tgtgaaagtgggtcttaagacgtcggtactgcatgtgacgcacgtagatcatgca*TTCACCGGTGGAGACGGTTTTCTTATAATGACACC*TAATTTCTACTCTTGTAGAT**ATGCTGATGCAACATAGGTTA**CAAATAAAACGAAAGGCTCAGTCGAAAGACTGGGCCTTTCGTTTTATcaacagcggtctactgaatctgagctagtgcgtgatataattaaaattatattca	Lower case for plasmid overlap and spacer, *italicized* for P_{dnaK} sequence, underlined for crRNA scaffold, **bold** for σ^{54} targeting sequence, Upper case for *rrnB*1 terminator	For CRISPRi knockdown of σ^{54}; insert into BamHI-digested pBOMBL12CRia (e.v.)::L2 plasmid
σ^{54} multiplexed crRNA	tgtgaaagtgggtcttaagacgtcggtactgcatgtgacgcacgtagatcatgca*TTCACCGGTGGAGACGGTTTTCTTATAATGACACC*TAATTTCTACTCTTGTAGAT**ATGCTGATGCAACATAGGTTA***taatttctact*	Lower case for plasmid overlap and spacer, *italicized* for P_{dnaK} sequence, underlined for crRNA scaffold, **bold** for σ^{54} targeting sequence, underlined and lower case for 2nd crRNA overlap	For multiplexed CRISPRi knockdown of σ^{54}; insert into BamHI-digested pBOMBL12CRia (e.v.)::L2 plasmid
σ^{28} multiplexed crRNA (overlaps σ^{54} targeting crRNA)	*tgcacataggtta*TAATTTCTACTCTTGTAGAT**CAAACTAAAAAATAAGGCTAA**CAAATAAAACGAAAGGCTCAGTCGAAAGACTGGGCCTTTCGTTTTATcaacagcggtctactgaatctgagctagtgcgtgatataattaaaattatattca	Lower case for plasmid overlap and spacer, underlined for crRNA scaffold, **bold** for σ^{28} targeting sequence, underlined and	For multiplexed CRISPRi knockdown of σ^{28} and σ^{54}; insert into BamHI-digested

		lower case for σ^{54} targeting crRNA overlap	pBOMBL12CRia (e.v.)::L2 plasmid
incA multiplexed crRNA	tgtgaaagtgggtcttaagacgtcggtactgcatgtgacgcacgtagatcatgca*TTCACCGGTGGAGACGGTTTTCTTATAATGACACC*TAATTTCTACTCTTGTAGAT**TCATTTAAAGTTCTCTGAAA***taatttctact*	Lower case for plasmid overlap and spacer, *italicized* for P_{dnaK} sequence, underlined for crRNA scaffold, **bold** for *incA* targeting sequence, underlined and lower case for 2nd crRNA overlap	For multiplexed CRISPRi knockdown of *incA*; insert into BamHI-digested pBOMBL12CRia (e.v.)::L2 plasmid

1. Ouellette SP, Blay EA, Hatch ND, Fisher-Marvin LA. 2021. CRISPR interference to inducibly repress gene expression in *Chlamydia trachomatis*. *Infect. Immun.* **89**: e0010821.
2. Reuter J, Otten C, Jacquier N, Lee J, Mengin-Lecreulx D, Löckener I, Kluj R, Mayer C, Corona F, Dannenberg J, Aeby S, Bühl H, Greub G, Vollmer W, Ouellette SP, Schneider T, Henrichfreise B. 2023. An NlpC/P60 protein catalyzes a key step in peptidoglycan recycling at the intersection of energy recovery, cell division and immune evasion in the intracellular pathogen *Chlamydia trachomatis*. *PLoS Pathog* **19**:e1011047.
